# Light force-powered cellular medical micromachines

**DOI:** 10.3389/fbioe.2026.1746261

**Published:** 2026-01-22

**Authors:** Dalin Ma, Xinyu Ren, Jiaxi Zheng, Linyue Zheng, Tong Yang, Hao Pang, Wei Chen, Zufang Lin, Xiaoshuai Liu

**Affiliations:** 1 Department of Optoelectronic Engineering, School of Physics and Materials Science, Guangzhou University, Guangzhou, Guangdong, China; 2 College of Electronic Engineering, South China Agricultural University, Guangzhou, China

**Keywords:** biophotonics, bioprinting, cellular micromachines, light force, optical tweezer

## Abstract

With the synergistic advancement of micro/nanotechnology and intelligent control systems, medical micromachines are emerging as promising alternatives to conventional diagnostic and therapeutic methods, offering enhanced operational precision and minimal invasiveness for precision medicine applications. However, most existing micromachines rely on artificial synthetic materials, which involve complex micro-nano fabrication and raise biosafety concerns regarding immunogenicity and limited long-term therapeutic efficacy in deep tissues. The integration of natural biological cells with programmable optical tweezer has opened new avenues to overcome these limitations, enabling precise behavioral regulation and *in situ* assembly of cell-based micromachines. This review systematically outlines the design strategies underlying five categories of light force-powered cellular micromachines, including chemotactic bacteria, photosynthetic microalgae, red blood cells (RBCs), immune cells and subcellular structures, and highlights their pioneering applications in targeted drug delivery, minimally invasive surgery and desired immunotherapy. Meanwhile, it also addresses key challenges such as limited tissue penetration depth, phototoxicity management and operation intelligence, while suggesting future directions like adaptive optics-driven swarm control, optomechanobiological coupling and bioprinting-integrated systems. Additionally, the convergence of photonic technology, synthetic biology and artificial intelligence is expected to advance these micromachines into next-generation biomedical platforms for health supervision and disease therapy *in vivo*.

## 1 Introduction

In contemporary medicine, the ability to perform real-time health monitoring, precise therapeutic interventions and targeted drug delivery is critical for enhancing treatment efficacy while minimizing therapy-related side effects (Meinders et al., 2025; Pan et al., 2023; Song et al., 2025). These capabilities substantially improve disease management and prognostic outcomes. To address these critical demands, medical micromachines, typically ranging in size from tens of nanometers to several micrometers, offer unique advantages by overcoming the dual limitations of operational precision and invasiveness trauma inherent in conventional diagnostic and therapeutic approaches (Sitti et al., 2015; Tay and Melosh, 2019). Their miniature scale allows them to traverse biological barriers in a minimally invasive manner and then navigate in complex physiological environments, including blood vessels, central nervous system, gastrointestinal tract and even dense tissue matrices (Huang et al., 2025). Moreover, they can achieve autonomous motion control, targeted positioning and anchoring as well as coordinated multifunctional operations (Ye et al., 2024), thus making them as promising platforms for executing multifunctional biomedical tasks, including real-time monitoring of pathological signals, targeted drug delivery, minimally invasive surgical operation and precise treatment of malignant tumors (Kong et al., 2023; Li et al., 2023; Parvin et al., 2025; Weerarathna et al., 2025; Zhou et al., 2023; Hossain et al., 2025).

Crucially, the progressive development of medical micromachines has been significantly accelerated by advances in micro/nano-fabrication, three dimensional (3D) printing and molecular self-assembly technologies (Liu J. et al., 2024; Sarabi et al., 2023; Wang H. et al., 2025). These technological breakthroughs not only improve the biocompatibility, motion controllability and environmental responsiveness of medical micromachines (Wang et al., 2021), but also establish their indispensable roles in early disease diagnosis and precise intervention of complex diseases (Ku et al., 2025). However, most reported medical micromachines are fabricated from artificial synthetic materials (such as resin (Davis et al., 2025) and hydrogel (Hu X. et al., 2024) and often require invasive delivery methods (e.g., injection) or, in the case of oral capsules (Abramson et al., 2019), face potential challenges related to gastrointestinal degradation and targeted release. More crucially, these artificial constructs often tend to trigger immune responses in the organism, leading to their rapid clearance before completing their intended medical tasks. This fundamental issue of biocompatibility and immune evasion has inspired the exploration of biologically derived alternatives (Niu et al., 2023). Capitalizing on innate biological advantages, cell-based medical micromachines not only evade host immune clearance but also preserve the innate biological functions of the source cells (Timin et al., 2018), such as the chemotactic navigation ability of bacteria, energy self-sufficiency of microalgae and immunoregulatory properties of immune cells (Alirezaeizanjani et al., 2020; Yu et al., 2024; Roe, 2024). The organic integration of native cellular capabilities with externally applied regulation is expected to enhance the biocompatibility of medical micromachines while unlocking dynamic diagnostic and therapeutic scenarios beyond the reach of conventional synthetic materials (Dogan et al., 2024; Li et al., 2024; Li Z. et al., 2025; Liu et al., 2019a; Li et al., 2019).

To exploit the full potential of cellular micromachines, various external field-based driving strategies, such as magnetic, electric, acoustic and optical fields, have been developed for precise navigation and remote control of cellular micromachines within complex biological environments (Wang Y. et al., 2023; Moon et al., 2023). Among these, optical tweezer has garnered considerable attentions due to its distinctive advantages (Xu et al., 2025), including non-contact operation, high biocompatibility, subcellular-scale resolution and seamless integration with real-time microscopy for visualization (Jamil et al., 2022; Pellicciotta et al., 2023). The synergistic combination of the innate biological intelligence of living cells with the high spatiotemporal precision of optical tweezer opens a promising pathway for developing advanced medical micromachines (Zhang and Liu, 2008), which not only enhance the desired biocompatibility but also unlock novel theragnostic capabilities (Favre-Bulle et al., 2019), such as real-time path correction for drug delivery and prolonged monitoring of disease microenvironments *in situ*, which remains challenging to achieve with traditional synthetic micromachines (Li X. et al., 2025; Wang Z. et al., 2025).

**FIGURE 1:**
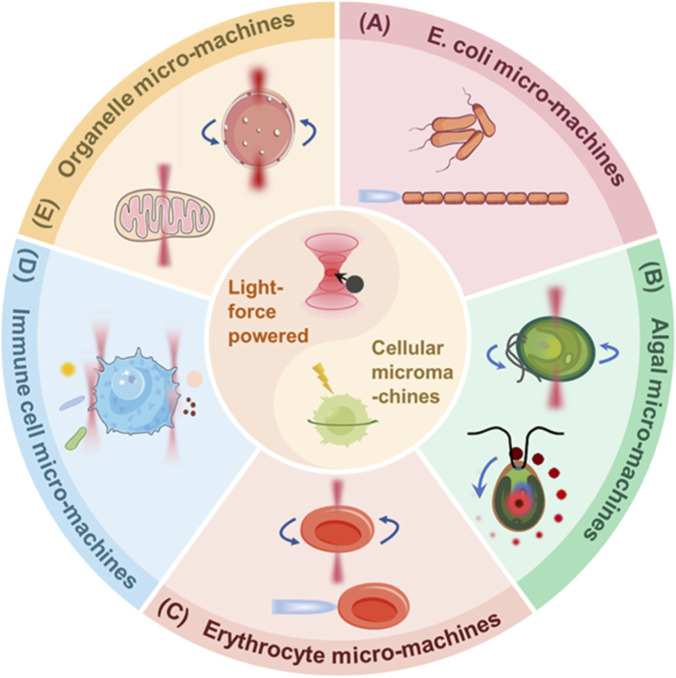
Schematic overview of light force-powered cellular medical micromachines constructed from five representative biological units. **(A)** Bacteria-based medical micromachines. Illustrated by *Escherichia coli*, optical forces enable precise spatial arrangement and chain-like assembly of bacterial cells, creating biological waveguides for signal transduction and biosensing. **(B)** Algae-based medical micromachines. Under optical trapping and rotational control with scanning optical traps (surrounding red spots), microalgae serve as multifunctional micromotors for targeted navigation, cargo delivery and biomanipulation in physiological environments. **(C)** RBC-based medical micromachine. Optical forces facilitate non-contact trapping and controlled rotation of RBCs, enabling their use as reconfigurable microlenses, microflow switches and signal transmission components. **(D)** Immune cell-based medical micromachines. Optical tweezers allow real-time control and *in situ* functional modulation of immune cells (illustrated by macrophages), supporting targeted drug delivery and active pathogen clearance. **(E)** Subcellular structure-based medical micromachines. Optical forces enable precise manipulation of organelles, such as the controlled rotation of cell nuclei to generate microfluidic flows and the directional transport of mitochondria for intercellular energy delivery.

In this review, we will systematically summarize the core design strategies and biomedical application of light force-powered cellular micromachines, constructed from five representative biological units including bacteria, microalgae, red blood cells (RBCs), immune cells, and subcellular structures (Figure 1). Emphasis is placed on leveraging the distinctive advantages of optical tweezer techniques, such as contactless actuation, high biocompatibility and subcellular spatial precision, to enable *in situ* assembly, directional migration and remote control of these cellular micromachines (Xu et al., 2025). Meanwhile, the review will explore their promising applications in targeted drug delivery, real-time *in vivo* diagnostics and precision immunotherapy. Finally, we analyze prevailing challenges and outline future research directions, with particular focus on harnessing the subcellular positioning accuracy and cross-scale manipulation compatibility of optical technologies for organized assembly and structural printing of natural biological components. Such advances are expected to pave the way for a new generation of light force-powered cellular micromachines with higher functional integration, enhanced physiological adaptability and superior therapeutic performance.

## 2 Physical principle of optical tweezer

Optical tweezer is a powerful technique that enables stable trapping and precise control of micro-/nanoscale objects by leveraging light–matter interactions (Grier, 2003; Ashkin et al., 1986). The core principle relies on a highly focused laser beam to exert finely controllable optical forces on targets such as biological cells (Ashkin et al., 1987), bacteria (Ashkin and Dziedzic, 1987), subcellular structures (Sparkes, 2018) and functional nanoparticles (Svoboda and Block, 1994), thus effectively serving as “invisible tweezers” for microscopic manipulation (Ashkin, 1970; Essiambre, 2021). The underlying mechanism involves focusing a laser beam into a diffraction-limited spot using a high numerical-aperture (NA) microscope objective (Farré and Montes-Usategui, 2010). When a small particle enters the focal region, momentum transfer from photons generates optical forces composed primarily of two components: optical gradient force and optical scattering force. The gradient force draws the microparticle toward the region of highest light intensity (i.e., the beam focus) with its magnitude proportional to the spatial intensity gradient of the laser beam. In contrast, the scattering force pushes the particle along the direction of light propagation due to radiation pressure while its magnitude is proportional to the light intensity. For stable optical trapping, the gradient force must dominate the scattering force which requires tightly focused and high-intensity laser beams to generate steep intensity gradients (Bradshaw and Andrews, 2017; Tamura et al., 2023).

Depending on the laser configuration, control mechanisms and underlying physical principles, optical manipulation techniques can be broadly classified into far-field and near-field methods. Far-field strategies, such as conventional optical tweezers (COTs) (Ashkin et al., 1986; Pesce et al., 2020), scanning optical tweezers (SOTs) (Volpe et al., 2023), holographic optical tweezers (HOTs) (Chen and Cheng, 2022) and optical fiber tweezers (OFTs) (Xin and Li, 2019; Zhao X. et al., 2020), rely on optical forces generated by a tightly focused laser beam in the far field to trap objects. In contrast, near-field strategies, including plasmonic optical tweezers (Ren et al., 2021) and evanescent wave optical tweezers (Zheng and Yu, 2024), exploit strongly enhanced optical gradients or momentum from confined light fields near surfaces to achieve trapping with potentially lower laser powers. Each variant is tailored to specific microscale manipulation scenarios, offering unique advantages in terms of spatial resolution, operation flexibility and system integration. Owing to their non-contact, high-precision and programmable characteristics, optical tweezers have been widely applied across biomedical field (Konyshev et al., 2021). Key applications include single-cell trapping and sorting (Keloth et al., 2018; Xu et al., 2023), real-time tracking of intracellular organelles (Zakrisson et al., 2016), mechanism manipulation and dynamic stretching of DNA molecules (Takahashi et al., 2021), microscale assembly for 3D bioprinting (Kirkham et al., 2015) and the design and actuation of medical micromachines (Palagi et al., 2016). The fundamental theories and operational mechanisms of optical tweezers have been extensively studied and are well-documented in the literature (Neto and Nussenzveig, 2000; Nieminen et al., 2007; Guo and Li, 2013). Therefore, they will not be discussed in detail here. Instead, this review will focus specifically on the application of optical tweezer techniques for constructing and actuating cell-based medical micromachines.

## 3 Light-force powered cellular medical micromachines

### 3.1 Bacteria-based medical micromachines

As naturally motile microorganisms, bacteria possess innate self-propulsion capabilities that enable precise navigation within complex microenvironments (Liu L. et al., 2020) and directional chemotaxis in response to chemical gradients (Patiño Padial et al., 2025). They also exhibit remarkable environmental resilience, allowing survival under extreme conditions, along with inherent biodegradability and excellent biocompatibility (Zhou, 2016; Liu et al., 2025). Moreover, in the near-infrared (NIR) wavelength range, bacteria display high optical transparency and possess a refractive index higher than that of their surrounding medium (Liu et al., 2014; Daher et al., 2022). These combined properties make them promising candidates as biological building blocks for constructing cellular biomedical micromachines.

**FIGURE 2:**
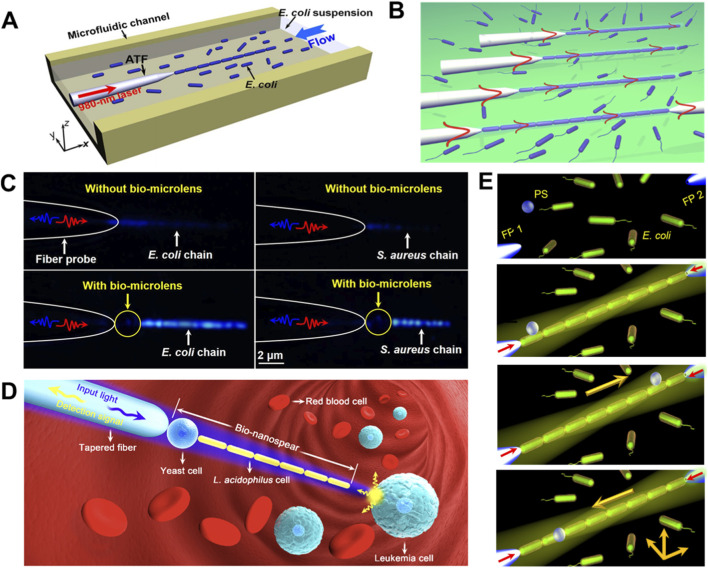
Light force-powered medical micromachines based on bacteria. **(A)** Schematic illustrating the optofluidic assembly of an *E. coli* cell chain. A tapered fiber probe integrated in a microchannel traps and sequentially aligns *E. coli* cells under 980 nm laser irradiation, forming stable chain structures for potential use as bio-optical waveguides (Xin et al., 2013a). **(B)** Schematic illustration for optical assembly of *E. coli* biophotonic waveguide. The sequential panels demonstrate waveguides of increased length, culminating in the connection of an output fiber for quantitative optical transmission measurement (Xin et al., 2013b). **(C)** Enhanced fluorescence and backscattering signals of bacterial chains via yeast-cell microlens. Comparative imaging shows *E. coli* and *S. aureus* chains without (upper) and with (lower) the microlens, where red and blue arrows indicate the 980 nm laser (trapping/excitation) and backscattering signal (bacterial counting), respectively (Li et al., 2017). **(D)** Schematic illustration of the living nanospear for near-field optical probing. This system enables precise scanning manipulation and localized fluorescence excitation/detection on leukemia cells in human blood samples (Li et al., 2018). **(E)** Schematic illustration of the bio-conveyor belt assembly and the bidirectional transport of a PS particle (Liu et al., 2019b).

In 2013, Xin et al. proposed an optofluidic strategy using an abrupt tapered optical fiber (ATF) to sequentially trap and assemble *Escherichia coli* (*E. coli*) cells into linear chain within a microfluidic environment (Xin et al., 2013a). By launching a 980-nm laser beam into the ATF, individual *E. coli* cells were successively trapped at the fiber tip and assembled into well-defined chain structures (Figure 2A). Meanwhile, the trapped cell number was increased from 2 to 17 as the laser power varying from 13 to 85 mW, and can suffer from a controlled shift in a microfluidic channel with a flow velocity of about 3 μm/s, suggesting their potential as functional bio-optical waveguides for studying bacterial communication, signal transduction and intercellular interactions. To validate this concept, they construct a biophotonic waveguide (bio-WGs) from the trapped *E. coli* cells, with a controlled length ranging from 4.6 to 54.5 μm (Xin et al., 2013b) (Figure 2B). Light propagation along these bio-WGs was visually confirmed, with the attenuation coefficient and propagation loss of 0.052 μm^−1^ and 0.023 dB/μm. Benefited from the constructed bio-waveguides, the signal transferred among the adjacent samples can be detected and monitored in real time, thus allowing to detect the quantitative response of the living biological samples to external stimuli without the need of remove the optical components. Further extending this work, Xin et al. fabricated branched *E. coli*-based bio-WGs while using a specially designed four-segment tapered fiber probe (Xin et al., 2015). These branched structures enabled multidirectional light propagation through the assembled branches I, II and III, with their branch length of 25.3, 27.7 and 23.2 μm and branch angles of 15°, 0° and13°, respectively. Moreover, the propagation loss for the three branches was obtained with results of 0.25, 0.23 and 0.26 dB/μm for branches I, II, and III, respectively. The proposed bacteria-based branched structure allows optical signals to be routed to multiple targets within biological systems, thus providing promising self-sustainable biphotonic devices such as multidirectional bio-waveguides and beam splitters. Moreover, as these waveguides are constructed entirely from natural biomaterials, they circumvent the biocompatibility limitations associated with exogenous synthetic materials, thereby advancing the prospects of bio-photonics for *in vivo* applications.

The successful demonstration of bacteria-based biophotonic waveguides established a foundation for using optically assembled cellular structures not just for signal transmission, but also for active light manipulation such as local reshaping and tight concentration of optical fields at the nanoscale. Building on this concept of cell-based photonics, research has explored using other microorganisms as intrinsic optical components. For instance, yeast cells have been exploited as natural bio-microlenses due to their spherical morphology and suitable refractive index, with the aim to generate photonic nanojets and thus enhance the fluorescence emission of upconversion nanoparticles (Figure 2C) (Li et al., 2017). Leveraging the photonic nanojet effect produced by the yeast cell, Li et al. confined excitation light into a subwavelength region and achieved a fluorescence enhancement of up to 100 times. These cellular microlens enabled single-cell fluorescence imaging of pathogenic bacteria such as *E. coli* and *Staphylococcus aureus* in dark-field experiments. Crucially, the measured signal-to-noise ratio was larger than 11 dB during *E. coli* trapping by the bio-microlens, thus enabling a sing-cell-resolution detection. However, the signal cannot be identified for the case without the biomicrolens, demonstrating a strong enhancement of the backscattering intensity can be achieved by the photonic nanojet generated by the microlens. With the unique advantages of high sensitivity, easy miniaturization, and excellent biocompatibility, the exhibited strategies might find potential applications in bioimaging, nanosensors and single cell analysis. Further integrating waveguiding and nanojet effects, Li et al. developed a living nanoprobe termed as “bionanospear” (Li et al., 2018) (Figure 2D). Benefited from sequential focusing by the TFP, yeat cell and *Lactobacillus acidophilus* cells, light propagating along this structure was concentrated into a spot with a full width at half maximum of 190 nm, enabling real-time near-field imaging and sensing at subwavelength resolution. Moreover, the bionanospear exhibited flexibility and deformability with minimal risk of puncturing or damaging the cell membrane even when it was forced against the leukemia cell and bent to an angle of 15°, positioning it as a noninvasive tool for bioimaging, nanosensing and single-cell analysis. In addition to waveguiding and microlens applications, the bacteria have been engineered as functional bio-conveyor belts for targeted transport. In 2019, Liu et al. constructed a biocompatible optical conveyor belt from *E. coli* cells using dual-fiber optical tweezers (Liu et al., 2019b) (Figure 2E). By leveraging evanescent waves generated onto the surface of optically trapped *E. coli* cells, the system achieved stable nanoparticle trapping and dynamic delivery. Meanwhile, bidirectional transport and controlled release of target particles were achieved by adjusting the laser power injected into two fiber probes. Compared to other optical conveyor belts that are constructed with metal or semiconductor materials, the proposed bio-conveyor belt exhibits the significant advantages including the easy fabrication, high flexibility, and better biocompatibility or biodegradability towards potential implementation *in vivo*, thus offering a noninvasive and biocompatible strategy suitable for biomedical assays, targeted drug delivers and biological nanoarchitectonics.

Overall, light force-powered cellular micromachines based on bacteria represent a promising platform for precision medicine, benefiting from their inherent biocompatibility, structural and functional adaptability and minimal invasiveness (Chen B. et al., 2024). However, several key scientific and technical challenges still impede their further clinical translation. A major limitation lies in functional instability, as bacterial motility and metabolic activity are highly sensitive to microenvironmental fluctuations in temperature (Moon et al., 2023), pH (Ratzke and Gore, 2018), and nutrient availability (Roller et al., 2016), leading to unpredictable behavior and compromised operational reliability. Besides, structural constraints, such as limited mechanical strength, modest propulsion efficiency and suboptimal optical responsiveness compared to synthetic microstructures, also restrict their precision and robustness in performing complex biomedical tasks. Biosafety remains another critical concern (Rommasi, 2022). Moreover, although bacterial micromachines exhibit good biocompatibility, their introduction as exogenous living entities carries risks of immune rejection or secondary infection, requiring thorough evaluation of long-term immunological impacts (Chen H. et al., 2025). Finally, moving from laboratory-scale demonstration to clinically viable production presents a significant bottleneck. While large-scale biomass production of bacteria in bioreactors is indeed a mature technology in the pharmaceutical industry, the challenge for bacterial-based micromachines lies in the scalable integration of post-cultivation functionalization and quality control processes (Ganeshan et al., 2021; Abdi et al., 2024). Specific hurdles include maintaining the functional stability and motility of engineered bacterial strains under mass culture conditions, achieving high-yield and uniform loading of therapeutic cargoes (e.g., drugs and nanoparticles), and ensuring consistent optical responsiveness or other engineered functionalities across vast populations of cells. These factors, rather than the mere amplification of cell numbers, lead to high batch-to-batch variability and limited functional reproducibility, which currently constitute the major barriers to standardized manufacturing and clinical translation.

### 3.2 Algae-based medical micromachines

In addition to bacteria, microalgae have gained significant attention as a natural biological resource for constructing multifunctional biomedical micromachines (Wang X. et al., 2025; He et al., 2025). As photosynthetic organisms, they possess specialized light-sensitive structures (Hegemann, 2008), such as the eyespot apparatus and thylakoid membrane system, which endow them with high sensitivity and specific responses to optical stimuli, offering a natural biomechanical foundation for precise light-driven actuation. In particular, their motile organelles like flagella or cilia allows them to exhibit autonomous and directed movement within complex biological microenvironments (Gilpin et al., 2020; Kage et al., 2024; Wei et al., 2024), with swimming efficiency and speed that substantially exceed those of traditional synthetic micromotors (Noselli et al., 2019; Lisicki et al., 2019). For example, flagellated microalgae such as *Chlamydomonas reinhardtii (CR)* can achieve high swimming speeds typically ranging from 95 to 110 μm/s (Xin et al., 2020; Zou et al., 2020), which substantially exceeds the propulsion speed of many traditional synthetic micromotors, often reported in the range of 1–9.8 μm/s under similar conditions (Liu et al., 2021; Liu L. et al., 2023). Moreover, microalgae exhibit low cytotoxicity, minimal immunogenicity and excellent biocompatibility (Shchelik et al., 2020), significantly reducing the risk of host immune rejection and systemic adverse reactions *in vivo*. Importantly, the algal cell surface is rich in reactive functional groups (e.g., hydroxyl and carboxyl groups) (Kerschgens and Gademann, 2018), and its intracellular architecture supports extensive modifiability, facilitating the precise loading and targeted delivery of therapeutic or diagnostic agents.

**FIGURE 3:**
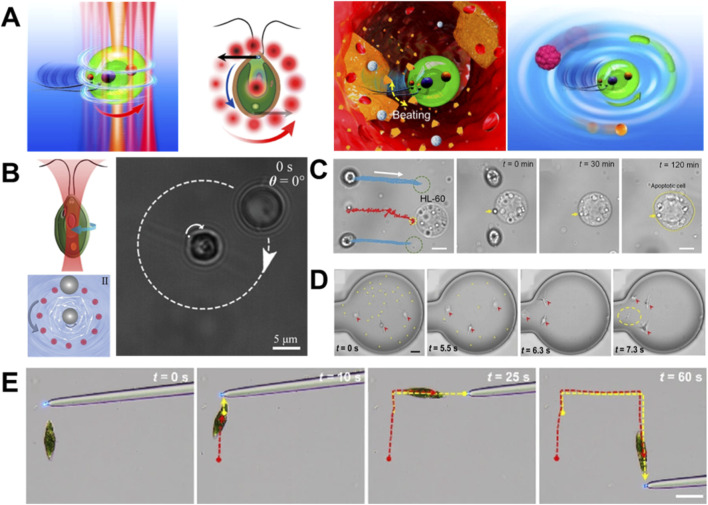
Light force-powered medical micromachines based on algae. **(A)** Schematic illustration of optically controlled micromotors based on a *CR* cell for flexible manipulation and targeted disruption of biological targets. The schematic shows a 3D view with a central trapping beam and an annular scanning path (curved arrow) to control micromotor rotation (panel I), a corresponding 2D layout with scanning optical traps (red spots, panel II), aggregate disruption by using flagella acting as manipulative arms (panel III), and microfluidic flow-driven target transport (panel IV) (Xin et al., 2020). **(B)** Schematics illustrating two rotation mechanisms of a CR cell including spontaneous rotation via a single-beam optical trap and micro vortex-driven rotation by viscous shear stress (Zou et al., 2020). **(C)** Biomicromotor tweezers for noninvasive cargo delivery and precision therapy. Two optically trapped CR cells generate hydrodynamic flows for targeted drug transport and controllable ablation of leukemia cells (Pan et al., 2022). **(D)** Time-sequence images of an OHD for non-invasive capture and removal of nano-biothreats. Yellow and red arrows indicate *E. coli* and the OHD, respectively (Xiong et al., 2023). **(E)** Controllable navigation of a photonic nanojet-regulated soft microalga robot. Red and yellow dashed curves mark the motion trajectories of the soft robot and the photonic nanojet, respectively (Xiong et al., 2024).

In 2020, Xin et al. developed a fully biological living micromotors constructed exclusively from *CR* microalgal cells, whose motion could be precisely regulated using optical forces (Xin et al., 2020) (Figure 3A). Capitalizing on the innate motility and phototactic behavior of *CR*, these micromotors exhibited efficient propulsion across diverse biological fluids, including cell culture, saliva, human serum, plasma, blood and bone marrow. In addition, they were capable of accomplishing complex biomedical tasks such as indirect manipulation of biological targets, controlled cargo transport and release as well as dynamic disruption of biological aggregates (e.g., blood clots *in vitro*). Furthermore, reconfigurable micromotor arrays enabled collaborative and high-throughput operations, highlighting the system’s scalability and functional versatility. This work represents a significant advance toward sustainable, intelligent and fully biological micromachine systems, showing considerable potential for *in-vitro* biomedical applications while circumventing limitations inherent to synthetic materials. In a complementary approach, Zou et al. developed a fully biocompatible cellular micromotor system by integrating optical tweezers with microfluidic vortex technique (Zou et al., 2020) (Figure 3B). Their approach utilizes a dynamically scanning optical trap following a circular trajectory to generate a localized microvortex, which imparts optically induced shear stress or torque for non-contact rotational manipulation of both motile and immotile cells. A key advantage of this method is its broad applicability as validated using nonmotile yeast cells and motile *CR* cells. The system also enables synchronized translational and rotational control, and can be scaled to form reconfigurable micromotor arrays. Owing to its high biocompatibility, operational safety and versatile control capabilities, this platform holds great potential for targeted drug delivery, real-time biosensing and *in vitro* therapeutic micromanipulation.

To address the challenges of active biological cargo delivery within complex biological microenvironments, Pan et al. proposed a noninvasive and fully biocompatible delivery platform based on “bio-micromotor tweezers” (Pan et al., 2022) (Figure 3C). This system employs two optically trapped *CR* microalgae cells as motile micromotors, generating localized hydrodynamic flow fields that enable contactless trapping, flexible manipulation and directional transport of various biological cargoes along programmable paths in diverse media. A key advantage of this approach is its ability to operate without direct physical contact or chemical conjugation, thereby preserving cargo integrity and minimizing biological damage. In a related advancement toward noninvasive manipulation, they developed an opto-hydrodynamic diatombot (OHD) for trapping and removing nanoscale biological threats such as viruses, *mycoplasma* and pathogenic bacteria (Xiong et al., 2023) (Figure 3D). By synergistically combining opto-hydrodynamic effects with optical trapping, the rotational OHD enables efficient collection of biological targets as small as 100 nm, allowing safe and dynamic elimination of nano-biohazards without compromising cellular viability. To further enhance operational adaptability in unstructured environments, Xiong et al. developed a soft microrobot based on *Euglena gracilis* microalgae which was regulated by a photonic nanojet (Xiong et al., 2024). In this system, a TiO_2_ microlens generates a photonic nanojet to precisely activate channelrhodopsin-2 (ChR2) in the microalga’s photoreceptor, thus transforming microalgae cell into a deformable soft microrobot (Figure 3E). By integrating the innate phototaxis with photonic nanojet-based optical stimulation, the proposed soft microalga microrobot enables subcellular-precision navigation through complex obstacles and facilitating targeted drug delivery at the single-cell level. This strategy might offer a novel biological tool for high-precision micromanipulation, demonstrating significant potential for therapeutic applications in dynamic and complex microenvironments.

### 3.3 Medical micromachine based on RBCs

Unlike bacteria and microalgae that typically require exogenous introduction, RBCs, as natural endogenous components of blood, possess inherent advantages including superior biocompatibility, minimal immunogenicity and high adaptability to the internal physiological environment (Chen et al., 2023). Their characteristic biconcave disc morphology and exceptional deformability enable smooth navigation through narrow capillaries and efficient distribution across bodily tissues (Ebrahimi and Bagchi, 2022). Moreover, RBCs can be functionally loaded with nanomedicines via membrane osmosis or surface modification strategies while maintain an extended circulation lifespan ranging from several weeks to months without requiring external energy sources (Jia et al., 2025). Recent advances have demonstrated that optical tweezers can achieve non-contact trapping, flexible manipulation and precise control of individual RBCs, establishing a robust technical foundation for their use in biomedical micromachines (Xie et al., 2022; Hu et al., 2022; Chen W. Y. et al., 2025; Zhu et al., 2020). These capabilities have spurred the development of RBC-based micromachines for diverse biomedical applications, such as optical imaging and detection as biomicrolenses (Miccio et al., 2015), active propulsion as micromotors (Wang K. et al., 2025), signal transmission as optical waveguides (Ahluwalia et al., 2010) and dynamic regulation as optofluidic switches (Liu X. et al., 2020) and microrouters (Liu X. et al., 2023). Such platforms provide versatile functionalities for biosensing, blood flow regulation and microenvironment monitoring, holding considerable promise for future clinical translation (Wang Q. et al., 2023; Hou et al., 2022).

**FIGURE 4:**
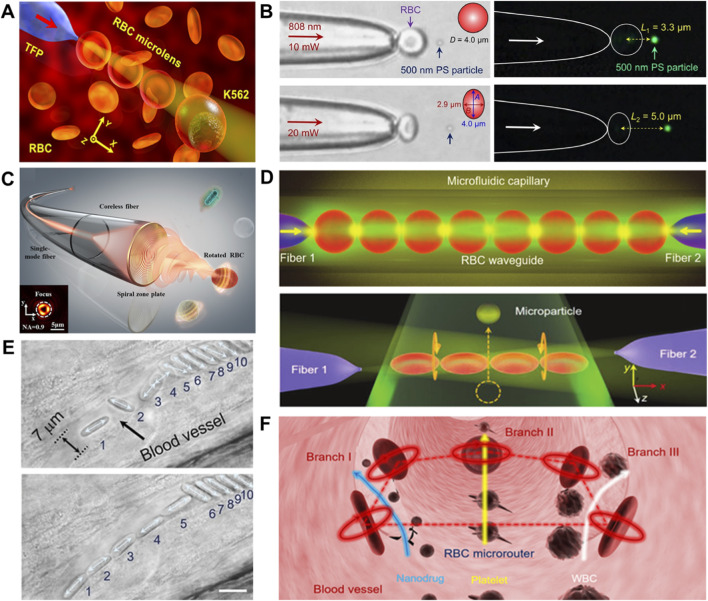
Light force-powered micromachines based on RBCs. **(A)** Schematic illustration of RBCs-based microlenses for single-cell membrane imaging and stretching (Liu et al., 2019c). **(B)** Microscope and fluorescence images demonstrating the optical trapping of a 500 nm fluorescent PS nanoparticle using tunable RBC microlens (Chen et al., 2022). **(C)** Schematic illustration of controlled RBC rotation via a single-fiber optical vortex tweezer. A functionalized fiber generates a tightly focused vortex beam for remote 3D manipulation and rotational control of RBCs (Wu et al., 2024). **(D)** Schematic illustration of an RBC waveguide assembled in a microfluidic capillary using dual optical fiber tweezers. The system also functions as an optically driven micromotor with controlled RBC rotation (orange arrows) enabling target microparticle transport (Li et al., 2019). (E) Reconfigurable optofluidic switch for blood microflow control *in vivo*. The micrographs demonstrate precise RBC positioning and dynamic shifting within a capillary. (Liu X. et al., 2020). (F) Optically programmable RBC microrouter for selective routing of biological targets *in vivo*. The schematic illustrates targeted guidance of different blood components into specific vascular branches under optical control (Liu X. et al., 2023).

In 2019, Liu et al. introduced an optical method using a TFP to assemble and manipulate RBCs into reconfigurable microlenses (Liu et al., 2019c) (Figure 4A). Not for the RBCs, the absence of intracellular organelles results in a relatively homogeneous intracellular refractive index, which was higher than the surrounding blood plasma (i.e., 1.40 vs. 1.33), thus enabling stable optical trapping and efficient light focusing. Meanwhile, their natural biconcave disc morphology inherently functions as a microlens element, and their remarkable deformability allows for dynamic tuning of optical properties, such as focal length under gentle optical forces. Moreover, RBCs are also suitable for constructing living biosensors to monitor and characterize potential blood disorders because their morphology is highly sensitive to the physiological properties of the local microenvironment. Unlike conventional substrate-fixed lenses, these RBC-based microlenses leverage these inherent opto-physiological properties to overcome rigidity constraints and enable precise 3D positioning. Using the mobile RBC microlens, they performed 3D scanning optical imaging of individual cell membrane with a magnification factor of 1.7, while simultaneously inducing controlled membrane stretching by a factor of 1.5. This method effectively combines high-resolution imaging with mechanical manipulation in a fully biocompatible and easily integrable platform, thus offering a versatile tool for biological imaging and studying cellular mechanical behaviors such as cancer metastasis. Building on this concept, Chen et al. developed a subwavelength-tunable biological microlens technology by leveraging optically controlled deformation of RBCs (Chen et al., 2022). By dynamically adjusting the laser power launched into a TFP, the system drives continuous deformation in swollen RBCs, i.e., transitioning from spherical to ellipsoidal and even flattened morphologies (Figure 4B). This controllable deformation enables precise tuning of the microlens focal length within the range of 3.3∼6.5 μm, facilitating subwavelength particle trapping at multiple positions and real-time modulation of fluorescence emission. Additionally, the platform achieved subwavelength imaging with continuously adjustable magnification from ×1.6 to 2×, highlighting its versatility in high-precision optical trapping and sensing applications.

Beyond RBC-based microlens, Wu et al. developed an optical micromotor for long-distance and rotational manipulation of RBCs (Wu et al., 2024) (Figure 4C). The system employs a functionalized optical fiber with a spiral zone plate (SZP) patterned on its end facet. With a numerical aperture of up to 0.9, the SZP generates a tightly focused vortex beam, enabling remote 3D manipulation and controlled rotation of target cells. In a complementary approach, Li et al. reported a living biosensor and micromotor based on an RBC-based optical waveguide (Li et al., 2019). The waveguide was formed by optically trapped RBCs between two TFPs (Figure 4D), enabling real-time pH sensing in the bloodstream through analysis of light propagation modes with a measurement accuracy of 0.05. Upon detecting abnormal pH levels, optical torque was applied to induce controlled rotation of the RBC waveguide, promoting local blood flow and targeted transport of microdrugs. This integrated platform offers a promising optofluidic manipulation strategy for early diagnosis of blood disorders and site-specific drug delivery.

In addition to serve as biological micromotors, RBCs have been engineered as label-free and biocompatible optofluidic switches for regulating blood microflow *in vivo* (Liu X. et al., 2020) (Figure 4E). By using SOTs to precisely arrange and rotate RBCs at vascular bifurcations, these optofluidic switches enable accurate control of blood microflow velocity and direction with a single-cell precision and rapid response time of approximately 200 ms. This noninvasive optofluidic strategy offers a powerful tool to probe the blood microenvironment and shows great potentials for studying and treating hematological disorders. Building on this concept, a living RBC-based microrouter has been developed through the organic integration of endogenous RBCs, programmable SOTs and flexible optofluidic strategy (Liu X. et al., 2023) (Figure 4F). By rotating five RBCs at controlled velocities and directions, a specific actuation microflow is achieved to exert the well-defined hydrodynamic forces on various blood components, thus enabling a selective routing by integrating dynamic input, inner processing and controlled output. Benefited from flexible programmability, it allows selective routing of multiple targets while towards different destinations, e.g., guiding platelets and white blood cells towards damaged vessel and cell debris for controlled hemostasis and targeted clearance, respectively. Moreover, it can also deliver large quantities of nanodrugs along predefined routes, offering a high-precision and programmable strategy for the active and targeted drug delivery *in vivo*.

Owing to their excellent biocompatibility and long circulation, RBCs represent an exceptional candidate for constructing cell-based micromachines. However, several critical challenges currently hinder their practical application. Among these, some are common to the broader field of light-force powered cellular micromachine, such as the limited penetration depth of light in deep tissues (Hu Y. et al., 2024), the instability of optical traps in high-flow-rate environments (Zhong et al., 2013), selective trapping of specific cells and multitarget parallel operation flexibility. Conversely, challenges more unique to RBC-based systems include ensuring the functional integrity and biomechanical stability of the RBC membrane during prolonged optical manipulation and cargo loading, and managing potential oxidative damage or morphological changes induced by prolonged laser exposure, which could affect their native circulatory lifespan and biocompatibility. Future efforts should focus on enhancing the robustness of optical trapping and developing novel strategies to overcome the above limitations. Crucially, the integration of artificial intelligence (AI) with biophotonics is expected to play a transformative role in advancing RBC-based micromachines. This convergence facilitates real-time decision-making through several practical applications: AI algorithms can process live imaging data for dynamic path planning, allowing micromachines to navigate complex vasculature while avoiding obstacles. Machine learning can optimize optical tweezer parameters in real-time to maintain stable control despite tissue scattering. Furthermore, AI-driven systems can correlate RBC biosensor data (e.g., pH or biomarkers) with therapeutic protocols to enable closed-loop, context-dependent actions, such as triggering precise drug release only at confirmed target sites. This shift from pre-programmed operation to adaptive intelligence significantly enhances therapeutic precision and efficacy in dynamic biological environments.

### 3.4 Immune cell-based medical micromachines

As core functional units of the body’s immune defense system, immune cells play a vital role in health monitoring, antigen clearance and immune homeostasis (Brancewicz et al., 2025; Marshall et al., 2024). Among them, neutrophils are primarily responsible for rapid pathogen detection and elimination, as well as modulation of inflammatory responses (Maier-Bega et al., 2024). Meanwhile, they serve as first responders during bacterial infection and tissue injury through phagocytosis, antimicrobial agents release and formation of neutrophil extracellular traps (Wang et al., 2024). Beyond neutrophils, macrophages represent another major class of innate immune effector cell with potent phagocytic activity (Chi et al., 2025). They contribute to host defense by eliminating pathogens and abnormal cells and play essential roles in inflammation resolution and tissue repair (Guan et al., 2025). Except for the neutrophils and macrophages, it is noteworthy that platelets, though traditionally recognized for their hemostatic functions, also exhibit important immunomodulatory roles. Beyond their well-established roles in blood coagulation and vascular repair, activated platelets undergo rapid morphological changes, upregulate surface adhesion molecules (Tan et al., 2025) and release a range of immunomodulatory factors (Everts et al., 2020; Martinez Bravo et al., 2024). These responses enable them to interact directly with pathogens, recruit and regulate leukocyte activity as well as participate in inflammatory processes. Such multifaceted immunoregulatory functions highlight platelets as critical players in bridging innate and adaptive immunity, extending their physiological significance beyond hemostasis to include active immune surveillance and modulation. Building on the functional diversity of these immune cells, the application of light force for real-time manipulation and *in-situ* construction of cellular medical micromachines not only deepen our understanding of immune behaviors but also open new avenues for clinical applications in targeted drug delivery and immunotherapy (Jeorrett et al., 2014; Zhao Q. et al., 2020).

**FIGURE 5:**
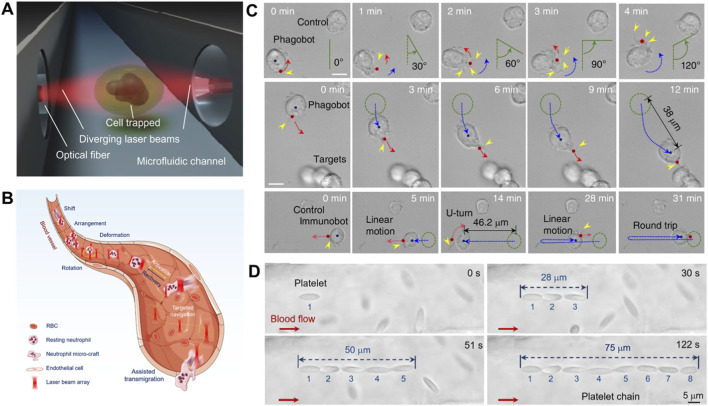
Light force-powered medical micromachines based on immune cells. **(A)** Schematic illustrating the principle of an optical stretcher. Counter-propagating laser beams exert controlled mechanical forces and enable contactless cellular morphometry and dynamic depolarization studies (Ekpenyong et al., 2017). **(B)** Schematic illustration of optically manipulated neutrophils serving as native microcrafts *in vivo*. The programmable optical force enable multiple cellular manipulations including stable trapping, directional shift, precise arrangement, dynamic activation, targeted navigation and assisted transmigration (Liu et al., 2022). **(C)** Motion control of the phagobot *in vitro*. Time-lapse sequences demonstrate controlled rotation along predefined trajectories (red dashed curve), targeted navigation toward specific cells (blue dashed line), and complex locomotor tasks integrating directional steering with U-turn maneuvers (Tan et al., 2025). **(D)** Optically reconfigurable platelet architecture with adjustable length *in vivo* (Qin et al., 2024).

In 2017, Ekpenyong et al. demonstrated that neutrophils can be primed via an optical stretcher and subsequently return to a quiescent state through oscillatory mechanical deformation (Ekpenyong et al., 2017) (Figure 5A). This mechanical modulation induced cellular depolarization at a rate two orders of magnitude faster than that observed in undisturbed cells. Building on these *in vitro* insights, Liu et al. successfully translated natural neutrophils into optically controlled biological microcrafts *in vivo* (Liu et al., 2022), which was achieved by integrating the innate immune functions of neutrophils with intelligent optical tweezer strategies (Figure 5B). The neutrophils were first optically trapped and then guided through controlled migration, spatial organization and real-time morphological transformation. A key mechanism involved periodic optical stretching to induce filopodia formation, which accelerated the transition from a resting state to activation state and allowed real-time steering of migration trajectories. This precise optical manipulation enhanced cell performance significantly, achieving a directed migration speed of 1.3 μm/s, i.e., approximately three times faster than spontaneous movement. Furthermore, these neutrophil-based micromachines were directed to perform complex tasks within physiological environments, including migration against blood flow, transmigration across vascular barriers and penetration deep into tissues. This work represents a major advance in cell-based micromachines, establishing a versatile platform for precision medicine applications such as targeted drug delivery, desired pathogen clearance and dynamic tissue repair, while also paving the way for emerging interdisciplinary fields including neurophotonics and immunophotonics.

In addition to neutrophils, macrophages play a pivotal role in immune responses and tissue homeostasis. Current strategies for macrophage-based microromachine design primarily leverage their innate phagocytic activity and immune-evasive properties to develop targeted drug delivery systems. Advancing this direction, Vasse et al. established an integrated platform combining optical tweezers, confocal fluorescence microscopy and microfluidics strategy to isolate individual macrophages and monitor their real-time responses to biochemical stimuli (Vasse et al., 2021). This contactless system overcomes key limitations of traditional cell-interaction studies, offering high spatiotemporal resolution for real-time observation of early macrophage activation. Such capabilities provide not only new insights into immune polarization mechanisms (Nguyen et al., 2023; Huo et al., 2025) but also a technical foundation for the precise construction (Chen and Hou, 2023; Nowak-Jary and Machnicka, 2024) and quantitative analysis of single-cell immune micromachines (Lin et al., 2024). Furthermore, Li et al. developed a light-powered phagocytic macrophage microrobot capable of precise navigation and targeted elimination of biological threats in both *in vitro* and *in vivo* settings (Figure 5C) (Tan et al., 2025). The system operates by optically activating resting-state macrophages with tightly focused NIR light, bypassing the need for genetic modification or artificial engineering. Upon activation, the macrophage microrobot exhibits guided directional motion via extended pseudopodia, enabling it to actively pursue and phagocytose diverse targets such as nanoplastics, microbes and cancer cell debris. Moreover, the approach was successfully demonstrated in live zebrafish, where endogenous macrophages were converted into functional micromachines, underscoring the system’s high biocompatibility and potential for immune modulation within complex physiological environments.

As natural biological glue, platelets play a vital role in hemostasis and vascular repair, exhibiting efficient intercellular and cell–matrix adhesion due to their rich membrane receptors and extracellular matrix proteins. These intrinsic properties make them ideal building blocks for constructing bio-micromachines with enhanced biocompatibility, targeted functionality, and physiological integration (Tang et al., 2020; Zhao et al., 2023). Capitalizing on these features, Qin et al. developed an optically reconfigurable platelet architecture *in vivo* by integrating programmable optical tweezer with the innate adhesive capacity of endogenous platelets (Qin et al., 2024) (Figure 5D). By precisely controlling the optical force landscape, multiple platelets were dynamically trapped, arranged, and spontaneously bound into stable microarchitectures without genetic or chemical modification. The resulting architectures demonstrated high flexibility and reconfigurability, enabling a series of biomedical functions such as obstacle circumvention, vessel labeling, flow regulation and cargo delivery within living vascular environments. This work establishes a novel strategy for creating multifunctional and modular micromachines from native cells, offering a promising platform for biomanufacturing, targeted drug delivery and advanced immunotherapies *in vivo*.

### 3.5 Subcellular structure-based medical micromachines

Subcellular structures play essential roles in cellular growth, development and stress response, maintaining homeostasis and function through highly coordinated molecular networks (Sparkes, 2018). Owing to their native size compatibility, intrinsic biocompatibility and functional specificity, endogenous subcellular structures have emerged as ideal building blocks for constructing multifunctional medical micromachines (Li et al., 2015). These natural components offer unique advantages for achieving subcellular-level precision in diagnosis and therapy, i.e., a level of control difficult to attain with traditional synthetic materials. Capitalizing on these properties, light forces have been employed to trap, arrange, and directionally transport subcellular structures with high precision. This capability enables controlled transfer and targeted delivery of functional components between different cells, allowing quantitative analysis of molecular mechanisms underlying cellular processes at the microscale. Such approaches not only deepen our understanding of intercellular coordination, signaling and transduction pathways but also facilitate the construction of multifunctional medical micromachines based on endogenous subcellular structures.

**FIGURE 6:**
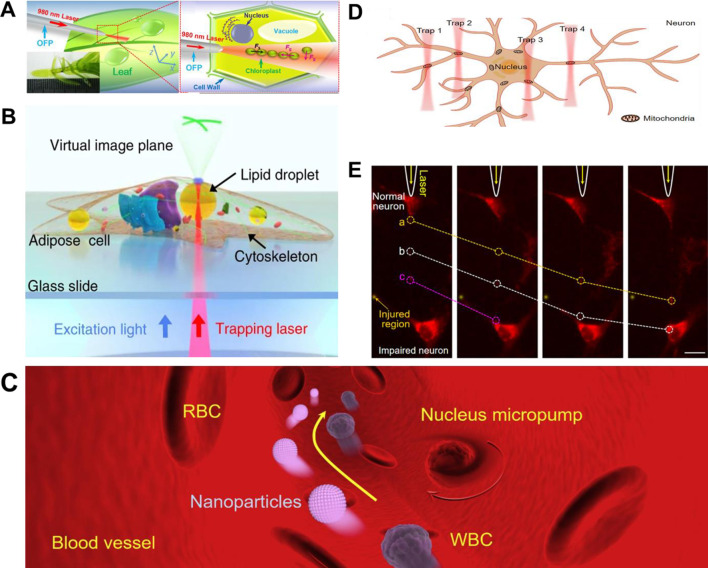
Light force-powered medical micromachines based on organelle. **(A)** Schematic illustration of intracellular binding of chloroplasts by using optical fiber tweezers (Li et al., 2015). **(B)** Lipid droplets as endogenous intracellular microlenses. An optically trapped droplet focuses light and collects fluorescence, enabling real-time imaging of subcellular structures and detection of extracellular signals (Chen et al., 2021). **(C)** Cell nuclei as endogenous micropumps. Optically controlled rotation of cell nuclei generates localized microfluidic field for enhanced nanoparticle and cell transport in blood vessels (Gao et al., 2021). **(D)** Optical manipulation and directional delivery of mitochondria within neurons (Gong et al., 2025). **(E)** Tunneling nanotubes as natural biophotonic conveyors for intercellular transport. Fluorescence imaging exhibits directional mitochondrial transfer from healthy to impaired neurons via optically guided nanotubes (Gong et al., 2024).

In 2006, Li et al. successfully demonstrated a non-contact optical tweezer strategy for intracellular binding and controllable manipulation of chloroplasts using an optical fiber probe (Li et al., 2015) (Figure 6A). By coupling a 980 nm laser beam into the fiber probe, stable optical trapping of chloroplasts within mesophyll cells was achieved, enabling rapid and efficient redistribution of the organelles as well as dynamic formation of chloroplast chains for the desired enhancement of photosynthetic efficiency. This optical strategy not only allowed controllable intracellular transport of chloroplasts but also achieved the precise assembly of two-dimensional chloroplast arrays, providing a novel methodology for studying organelle interactions, intracellular signaling and subcellular spatial organization. Building on the optical trapping of organelles, subsequent research has expanded to include other functional subcellular structures. In 2021, Chen et al. demonstrated the intracellular lipid droplets can work as inherently biocompatible microlenses (Chen et al., 2021), capable of focusing excitation light in a non-contact mode and enhancing fluorescence signals upon contact mode (Figure 6B). These lipid-based microlenses could be precisely positioned using light force, enabling real-time imaging of subcellular components and detection of extracellular signals. This strategy successfully enhanced the fluorescence visualization of cellular elements, including the cytoskeleton, lysosomes and adenoviruses, while reducing the required excitation power by up to 73%. In a complementary approach, Gao et al. proposed a light-driven endogenous micropumps *in vivo* (Gao et al., 2021) that was constructed using cell nuclei derived from RBCs (Figure 6C). Under programmable optical force generated by SOTs, the trapped cell nucleus underwent controlled rotation, producing localized microflows capable of transporting nanoparticles and cells within confined microvascular environments. In contrast to traditional synthetic micromachines, these native systems exhibit superior biocompatibility and operational flexibility. Their ability to operate within microliter-scale blood samples and accomplish multiplexed transport highlights their promise for target drug delivery, particularly in the context of hematological disorders.

Given their central role in neuronal metabolism and signaling, mitochondria have emerged as promising candidates for functional microscale interventions in neurodegenerative diseases. Their intrinsic biocompatibility and fundamental position in cellular energy pathways make them particularly suitable as endogenous micromachines for biomedical applications. Using SOTs combined with fluorescence imaging, Gong et al. have achieved precise and directional manipulation of individual mitochondria within neuronal axons (Gong et al., 2025) (Figure 6D). This methodology enables targeted mitochondrial positioning and transport, offering new avenues for subcellular regulation and therapeutic innovation in neurological disorders. Expanding the scope of organelle-level control, they further utilized tunneling nanotubes (TNTs) as natural biophotonic conveyors for intercellular organelle transport (Gong et al., 2024) (Figure 6E). In details, these membrane nanotubes can function as nanoscale optical waveguides, capable of guiding NIR light to generate optical forces for directional transport of mitochondria and nanovesicles between living cells. Notably, this approach has been applied to prevent mitochondrial hijacking from immune cells to cancer cells, thereby reactivating immune responses and suppressing tumor progression. Moreover, the technique operates without requiring genetic modification or pharmacological treatment, enabling the study of intercellular communication under physiological conditions. By leveraging native cellular structures as biophotonic conveyors, this approach provides a minimally invasive platform with superior biocompatibility and multifunctional capabilities, showing significant potential for future clinical applications in targeted therapy and cellular manipulation.

Subcellular structures, serving as fundamental units enriched with diverse functional components, constitute the structural basis for cellular homeostasis (Surovtsev and Jacobs-Wagner, 2018; Ding et al., 2025). Their inherent biocompatibility, reproducibility and relative ease of isolation make them attractive candidates for biomedical applications. Dynamic manipulation of these structures not only advances our understanding of cellular physiology but also facilitates the development of biologically derived, highly compatible components for next-generation medical micromachines. However, the high complexity of the intravital microenvironment presents substantial challenges to the stability and functional robustness of subcellular structures. Achieving precise manipulation and real-time responsiveness under such conditions remains a critical hurdle. Current limitations include insufficient capacity for coordinated regulation of multiple subcellular targets, low operational throughput and significant barriers to clinical translation. Looking forward, interdisciplinary advances integrating materials science, optical engineering and AI are expected to equip subcellular structures with programmable functionalities for desired clinical diagnostics and therapeutics *in vivo*, which might accelerate subcellular-level precision medicine in clinical practice.

## 4 Challenges and future

In this review, the recent advances and design principles were reported for five major categories of cellular micromachines driven by optical forces, highlighting their applications in targeted drug delivery, real-time diagnostics, single-cell surgery and immunotherapy. To offer an integrated perspective and facilitate direct comparison, the key characteristics, including cell size, experimental media, typical laser power requirements, optical tweezer configurations and reported biosafety considerations, were summarized in Table 1 for the primary classes of bio-micromachines discussed herein. This synthesis of quantitative parameters offers a foundational perspective for assessing the technological landscape, which will inform the subsequent discussion of prevailing challenges and promising future research avenues.

**TABLE 1:** Detailed performance comparison of Light force-powered cellular medical micromachines.

Type	Cell	Size (shape)	Experimental media	Laser power	Type of OT	Biosafety	References
Bacteria	*E. coli*	Length: 0.8–5 μm; Diameter: 440–540 nm (rod shape)	*In vitro*: cell culture medium	980 nm; 15–120 mW	OFT	No observable photodamage	Xin et al. (2013a), Xin et al. (2013b), Xin et al. (2015), Liu et al. (2019b)
	Yeast cell	Radius: 1.0 ± 0.4 μm (spherical shape)	*In vitro*: cell culture medium	980/808 nm; Less than 200 mW	OFT	No observable photodamage	Li et al. (2017), Li et al. (2018)
Algae	Chlamydomonas reinhardtii	Length: 6.2–6.7 μm; Diameter: 4.2–4.3 μm (spherical shape)	*In vitro*: cell culture medium	1,064 nm 0–150 mW	SOT	Biocompatibility and biodegradability	Xin et al. (2020), Zou et al. (2020), Pan et al. (2022)
	Diatom	Length: 7.9 μm; Diameter: 1.8 μm (spindle shape)	*In vitro*: cell culture medium	1,064 nm 50 mW	SOT	High biocompatibility	Xiong et al. (2023)
	Euglena gracilis	Length: 50 μm; Diameter: 10 μm (spindle shape)	*In vitro*: culture medium	478 nm 12 mW	OFT	High biocompatibility	Xiong et al. (2024)
Erythrocyte	Red blood cell	Diameter: 6–8 μm (Disk shape)	*In vitro*: cell culture medium; *In vivo*: living larval zebrafish	808/980/1,064 nm; 1–300 mW	OFT/SOT	High biosafety and biocompatibility	Li et al. (2019), Liu X. et al. (2020), Liu X. et al. (2023), Liu et al. (2019c), Chen et al. (2022), Wu et al. (2024)
Immunocyte	Neutrophil	Diameter: 8–12 μm (spherical shape)	*In vitro*: cell culture medium; *In vivo*: living zebrafish	1,064 nm; 50–200 mW	OFT/SOT	High biocompatibility	Ekpenyong et al. (2017), Liu et al. (2022)
	Macrophage	Diameter: 10–15 μm (spherical shape)	*In vitro*: cell culture medium; *In vivo*: living larval zebrafish	1,064 nm; 20–80 mW	OFT/SOT	High biocompatibility	Tan et al. (2025), Vasse et al. (2021)
	Platelet	Length: 8.5 μm; Width: 3.7 μm	*In vivo*: living zebrafish	1,064 nm 50–200 mW	SOT	High biosafety	Qin et al. (2024)
Subcellular structure	Chloroplast	Major axis: 2.3 μm; Minor axis: 1.2 μm (ellipsoidal)	*In vivo*: Hydrilla verticillate leaf cell	980 nm 30–65 mW	OFT	Undamaged	Li et al. (2015)
	Lipid droplet	Diameter: 0.5–40 μm (spherical shape)	*In vivo*: intracellular	1,064 nm; 1.5–10.8 mW	SOT	High biocompatibility	Chen et al. (2021)
	Cell nucleus	Diameter: 2.4 μm (spherical shape)	*In vivo*: zebrafish blood vessel	1,064 nm 50–200 mW	SOT	Fully biocompatible	Gao et al. (2021)
	Mitochondrion	Length: 0.5–2 μm; Diameter: 0.1–0.5 μm (rod/oval shape)	*In vivo*: neuron cell	1,064 nm 6–100 mW	OFT/SOT	Undamaged	Gong et al. (2025), Gong et al. (2024)

While significant progress has been made in the development of light force-powered bio-micromachines, most studies discussed in this review remain focused on *in vitro* systems. Nonetheless, a number of efforts have successfully extended toward *in vivo* applications. Benefited from the unique capability of non-contact, remote and highly precise manipulation, optical tweezers have shown great promise for potential translation in living biological environments. For example, by employing an oil-immersion objective to achieve extended focal depth (∼40 μm), Zhong et al. demonstrated 3D trapping of individual RBC within live murine ear capillaries using optical tweezers, and achieved a controlled restoration of blood flow by removing vessel-occluding cells (Zhong et al., 2013). In a similar way, Johansen et al. demonstrated precise manipulation of diverse blood cells (RBCs and macrophages) and microinjected bacteria in zebrafish embryos, achieving parallel trapping and programmable patterning of multiple nanoparticles to quantitatively analyze their adhesive interactions with vascular endothelia *in vivo* (Johansen et al., 2016). Moreover, Liu et al. reported a programmable optical tweezer strategy that enables real-time, active trapping and targeted delivery of nanodrugs *in vivo*, achieving target therapy of individual leukemia cell and cerebral thrombi within living vasculature (Liu X. et al., 2024). In a separate investigation, the same group remotely activated neutrophils and guided them along designated routes *in vivo*, enabling them to perform specific tasks such as active intercellular connections, targeted delivery of nanomedicine and precise elimination of cell debris, without requiring additional modification of the native neutrophils (Liu et al., 2022). Furthermore, Favre-Bulle et al. developed a dual-trap optical technique for precise manipulation of otoliths in live zebrafish, thus generating fictive vestibular stimuli in stationary zebrafish to elicit compensatory tail corrective movements and ocular counter-rolling in both eyes (Favre-Bulle et al., 2017).

Despite these advances, translating and implementing such technologies *in vivo* remains challenging. One major constraint lies in their limited adaptability to complex biological environments. The absorption and scattering of light by biological tissues restrict optical penetration to superficial layers at the millimeter scale, hindering their access to deep-seated lesions. Furthermore, the dynamic nature of the blood flow environment interferes with navigation accuracy and propulsion efficiency, substantially compromising the stability and flexibility of optical control. Target recognition, selective capture and manipulation of specific cells are also difficult. The intravital microenvironment is highly heterogeneous, encompassing diverse cell populations with variable phenotypes and functions. Individual cells contain subcellular structures and biomacromolecules that differ in size, morphology and optical properties, often distributed nonuniformly. This pronounced heterogeneity can introduce nonspecific interactions and background interference, reducing the targeting accuracy and manipulation selectivity of optical tweezers. In terms of manipulation capability, a clear throughput bottleneck exists in multi-target parallel operation. Most conventional optical tweezer systems support only single-beam or limited multi-beam control, rendering them inefficient for large-scale tasks, such as sorting hundreds of circulating tumor cells in blood. While HOTs can generate multiple optical traps simultaneously, they introduce significant system complexity and suffer from mutual interference among adjacent optical traps. Functionally, current light force-driven cellular micromachines remain relatively limited in capability and cannot yet achieve integrated imaging, diagnosis, therapy and feedback in a continuous process. From a safety perspective, potential phototoxicity remains a concern. Although systems built from endogenous biological materials avoid the immunogenicity risks associated with synthetic components, new challenges emerge. For example, bacteria may trigger immune reactions or uncontrolled proliferation while the photosensitivity of microalgae could disrupt normal physiological functions, which may ultimately impact therapeutic efficacy and biosafety.

To address the above challenges, integrated interdisciplinary strategies are essential. In the field of biophotonics, limitations in light–tissue interactions may be mitigated through advanced optical designs and control methods. The use of biocompatible NIR lasers, combined with precise modulation of optical field distribution, can help reduce phototoxicity while preserving manipulation efficacy (Khan et al., 2015). Furthermore, exploiting effects such as photonic nanojets can enhance localized optical field intensity, thereby improving control precision (Chen et al., 2004). The incorporation of adaptive optics, particularly through spatial light modulators that detect and dynamically compensate for wavefront distortions, enables accurate optical control even in deeper tissue regions (Rodríguez and Ji, 2018; Yoon et al., 2022). Emerging hardware advances, such as multimode optical fibers (Čižmár and Dholakia, 2011) and miniaturized implantable lens probes (Park et al., 2025), can physically deliver light into deep tissues, thereby effectively overcoming the penetration limits of superficial optical systems. Looking forward, integration with photoacoustic imaging may offer a pathway for precise and noninvasive manipulation of cells in deep-seated areas, providing a robust platform for cell-level diagnosis and therapy (Chen Z. et al., 2024; Daohuai et al., 2023).

Precise target recognition, capture and manipulation can be achieved through multidimensional strategies. First, morphology-based preselection leverages the ability of optical tweezers to focus laser beams to submicrometer dimensions. When combined with high-resolution microscopy, this approach enables accurate localization and selective trapping of single cells or specific subcellular structures with distinctive morphological features. Second, the integration of AI assisted recognition algorithms provides a powerful complementary strategy. By constructing comprehensive databases of morphological and optical characteristics across cell types, as well as employing deep learning models for feature extraction and fine-grained image classification, both the efficiency and specificity of cell identification can be substantially enhanced. Such AI-driven frameworks further facilitate intelligent trap loading and real-time modulation of optical potentials, enabling robust single-cell-level capture and manipulation within complex biological environments.

In the field of intelligent control, the advancement of large-scale parallel optical tweezer systems is crucial. By integrating deep learning-driven optimization algorithms, it becomes feasible to generate hundreds or even thousands of independently controllable optical traps, enabling high-throughput cell manipulation and dynamic sorting. Furthermore, the seamless integration of optical tweezer with microfluidic technologies offers a synergistic strategy: microfluidics can perform preliminary coarse positioning and screening, while optical tweezers provide subsequent precise capture and remote actuation. This combined approach significantly enhances the assembly efficiency of light force-powered cellular micromachines and extends their utility in large-scale biomedical applications. In parallel, AI can be leveraged for efficient data processing and real-time feedback control, allowing dynamic optimization of micromachine trajectories and operational tasks. Together, these intelligent control strategies substantially improve the adaptability and functional performance of such systems in complex and dynamic biological environments.

**FIGURE 7:**
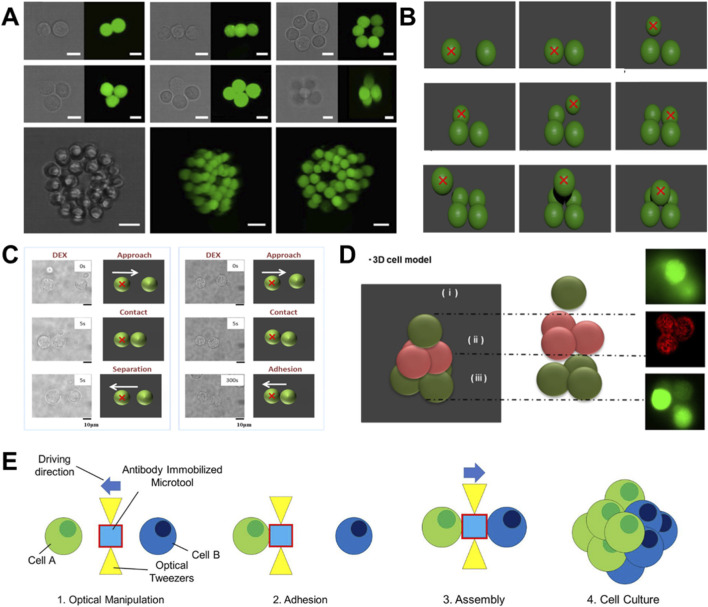
Cellular aggregates created by 3D bioprinting. **(A)** Brightfield and fluorescent confocal (green-labeled) images illustrating the precise assembly of complex cellular microstructures using HOTs (Kirkham et al., 2015). **(B)** Schematic illustrating the stable pyramidal assembly of epithelial cells in PEG-containing medium via optical tweezers (Hashimoto et al., 2016). **(C)** Optical manipulation of a pair of epithelial cells in the presence of dextran for 5 s or 300 s (Yoshida et al., 2017). **(D)** Schematic and representative micrographs illustrating an MS1 cell layer sandwiched between adipose-derived mesenchymal stem cells, constructed via optical tweezer in a DEX solution (Yamazaki et al., 2019). **(E)** Optical trapping and assembly of cell culture using an antibody-functionalized microtool (Mori et al., 2023).

In the field of biomanufacturing, the integration of 3D bioprinting technology enables high-precision spatial assembly of cells and subcellular structures with diverse functionalities, thereby facilitating the efficient fabrication of advanced microscale medical machines. More significantly, this approach offers the capability for on-demand construction and controlled disassembly of these systems, enabling the creation of customized and functionally enhanced micromachines with improved adaptability. Recent studies have validated the feasibility of 3D bioprinting for spatially controlled cell assembly, successfully generating organized cellular aggregates and providing a viable pathway toward developing more sophisticated biomedical systems in the future. In 2015, Kirkham et al. presented a HOTs-based micromanipulation platform capable of constructing 3D cellular micro-architectures with sub-micron precision (Kirkham et al., 2015) (Figure 7A). By integrating embryonic stem cells, extracellular matrix components, hydrogels and controlled-release microparticles, they have successfully engineered biomimetic microenvironments that recapitulate key structural and biochemical characteristics of native stem cell niches. This integrated strategy enables spatiotemporal control over cellular organization and chemical signaling while supporting accurate reconstruction of multicellular architectures *in vitro*. The resulting platform offers unprecedented resolution and complexity for investigating cellular behaviors, developmental dynamics and microenvironmental regulation. Although optical tweezers technology can perform non-contact manipulation of cells, it is difficult to maintain stable intercellular contact. Conventional approaches often rely on xenobiotic linkers, such as hydrogels, avidin-biotin systems or chemically modified surfaces, to stabilize cell–cell connections, which may introduce cytotoxicity and impair normal cellular functions. To address this limitation, Hashimoto et al. developed a scaffold-free strategy for constructing stable 3D cellular assemblies using light force while within a macromolecular crowding environment (Hashimoto et al., 2016) (Figure 7B). By introducing a hydrophilic polymer such as polyethylene glycol (PEG), they demonstrated that transient optical pairing of cells can trigger sustained cell–cell adhesion that persists even after polymer removal. This effect is attributed to depletion-induced interactions and membrane reorganization, wherein nanoscale proximity between opposing membranes promotes stable adhesion through thermally driven interactions while preserving membrane fluidity. Notably, the technique causes no observable cellular damage and offers a new avenue for scaffold-free tissue assembly. Preliminary findings also suggest that natural polymers such as dextran and albumin may serve as alternatives to synthetic PEG, highlighting the translational potential of this approach in tissue engineering and regenerative medicine. In 2017, Yoshida et al. introduced dextran, a natural hydrophilic polymer, as a more biocompatible and less invasive crowding agent (Yoshida et al., 2017) (Figure 7C). Using dextran to induce macromolecular crowding, they successfully achieved robust cell–cell adhesion via depletion interactions, and highlights the central role of the depletion effect rather than osmotic pressure in promoting direct attractive interactions between cells. In addition, they demonstrated that both dextran and PEG can elicit similar cell assembly behavior despite their structural differences, thus offering a promising platform for single-cell manipulation with broad implications for regenerative applications.

Building upon advances in optical trapping of biological cells, Yamazaki et al. successfully constructed 3D cellular assemblies using adipose-derived mesenchymal stem cells and endothelial cells, demonstrating the potential for creating complex tissue-like structures (Yamazaki et al., 2019) (Figure 7D). In parallel, Mori et al. established a novel microfluidic platform that enables single-cell-level assembly of cellular clusters through optically driven and antibody-functionalized microtools (Mori et al., 2023) (Figure 7E). They functionalized SU-8 microstructures with specific antibodies targeting membrane proteins CD51/61 and CD44, allowing selective cell trapping via antigen-antibody interactions. When manipulated by light force within the microfluidic environment, these functional microtools achieved precise trapping, directional transport and controlled assembly of HeLa cells into predefined clusters. Comparative validation confirmed the superior adhesion performance of antibody-coated microtools over non-functionalized counterparts. This integrated approach provides a versatile and modular strategy for bottom-up construction of sophisticated multicellular systems, offering significant potential for applications in tissue engineering, regenerative medicine, and high-throughput drug screening platforms. These advances demonstrate that optical tweezer-based 3D bioprinting enables precise fabrication of desired cellular aggregates, which can serve as effective “seeds” for generating tissue-like assemblies with biologically relevant compositions. The resulting constructs provide foundational units for *in vitro* organ modeling, establishing a key technological platform for biomedical research. Importantly, as highlighted earlier, optical tweezers offer unique flexibility in manipulating diverse subcellular structures and functional cell types (Moffitt et al., 2008). By integrating the multifunctional capabilities of these natural biological components, it becomes feasible to dynamically assemble light force-powered cellular microrobots in real time with the aim to satisfy specific therapeutic requirements (Hu et al., 2022).

## 5 Conclusion

This review has systematically summarized five major categories of medical micromachines constructed from natural biological components, with a specific focus on four native cell types, i.e., bacteria, microalgae, red blood cells and immune cells, along with various subcellular structures as distinct functional units. We have highlighted the unique biological properties of each cellular and subcellular component and explored how these inherent characteristics can be leveraged to design specialized micromachines for biomedical applications. The potential applications of these systems span multiple areas, including fundamental capabilities such as optical imaging, sensing and detection, as well as advanced clinical functions encompassing real-time diagnosis, targeted drug delivery, and desired disease treatment. This research direction not only provides diverse biological tools for precision medicine but also significantly advances the practical implementation of medical micromachines in disease management. Furthermore, we have addressed the multidimensional challenges hindering clinical translation and proposed potential design optimization strategies. The integration of light-controlled 3D bioprinting technologies enables on-demand and real-time fabrication of functionally sophisticated cellular micromachines, thereby establishing a robust technological foundation for future precision medicine applications. In summary, cellular medical micromachines represent both a promising therapeutic modality in biomedicine and a core technological platform emerging from deep interdisciplinary integration, which will accelerate the development of personalized and intelligent medical approaches as well as hold substantial potential to drive transformative advances in clinical diagnosis and treatment.
